# 5-(5-Bromo-2-meth­oxy­phen­yl)-2-fluoro­pyridine

**DOI:** 10.1107/S1600536812034435

**Published:** 2012-08-08

**Authors:** Muhammad Adeel, Fazal Elahi, M. Nawaz Tahir

**Affiliations:** a Department of Chemistry, Gomal University, Dera Ismail Khan, K.P.K., Pakistan; b Department of Physics, University of Sargodha, Sargodha, Pakistan

## Abstract

In the title compound, C_12_H_9_BrFNO, the dihedral angle between the aromatic rings is 51.39 (5)°; the C atom of the meth­oxy group is close to being coplanar with its attached ring (r.m.s. deviation = 0.0172 Å] and is oriented away from the pyridine ring. In the crystal, mol­ecules inter­act by van der Waals forces.

## Related literature

For a related structure, see: Adeel *et al.* (2012 ▶); Elahi *et al.* (2012 ▶ *a ▶,b* ▶); Elahi *et al.* (2012 ▶).
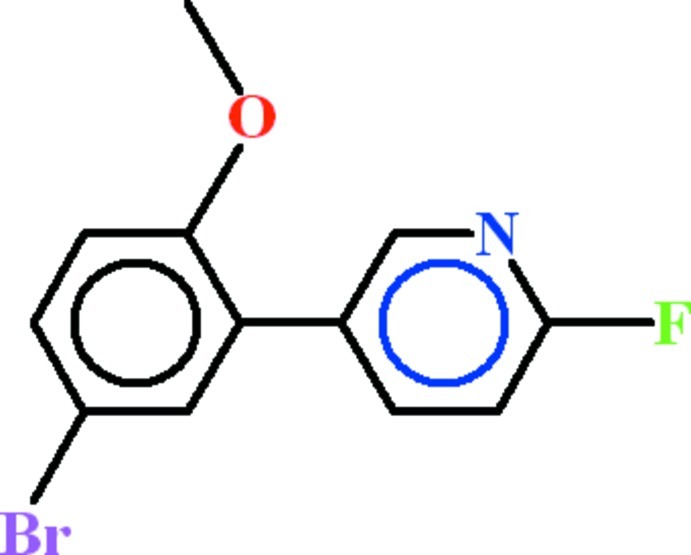


## Experimental

### 

#### Crystal data

C_12_H_9_BrFNO
*M* *_r_* = 282.11
Monoclinic, 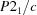

*a* = 3.9376 (4) Å
*b* = 20.999 (3) Å
*c* = 13.2700 (15) Å
β = 95.035 (7)°
*V* = 1093.0 (2) Å^3^
*Z* = 4
Mo *K*α radiation
μ = 3.75 mm^−1^
*T* = 296 K
0.34 × 0.18 × 0.16 mm

#### Data collection

Bruker Kappa APEXII CCD diffractometer
Absorption correction: multi-scan (*SADABS*; Bruker, 2005 ▶) *T*_min_ = 0.674, *T*_max_ = 0.698
7773 measured reflections
2027 independent reflections
1441 reflections with *I* > 2σ(*I*)
*R*_int_ = 0.042

#### Refinement

*R*[*F* ^2^ > 2σ(*F* ^2^)] = 0.035
*wR*(*F* ^2^) = 0.068
*S* = 1.04
2027 reflections
146 parameters
H-atom parameters constrained
Δρ_max_ = 0.29 e Å^−3^
Δρ_min_ = −0.28 e Å^−3^

### 

Data collection: *APEX2* (Bruker, 2007 ▶); cell refinement: *SAINT* (Bruker, 2007 ▶); data reduction: *SAINT*; program(s) used to solve structure: *SHELXS97* (Sheldrick, 2008 ▶); program(s) used to refine structure: *SHELXL97* (Sheldrick, 2008 ▶); molecular graphics: *ORTEP-3 for Windows* (Farrugia, 1997 ▶) and *PLATON* (Spek, 2009 ▶); software used to prepare material for publication: *WinGX* (Farrugia, 1999 ▶) and *PLATON*.

## Supplementary Material

Crystal structure: contains datablock(s) global, I. DOI: 10.1107/S1600536812034435/hb6919sup1.cif

Structure factors: contains datablock(s) I. DOI: 10.1107/S1600536812034435/hb6919Isup2.hkl

Supplementary material file. DOI: 10.1107/S1600536812034435/hb6919Isup3.cml

Additional supplementary materials:  crystallographic information; 3D view; checkCIF report

## Figures and Tables

**Fig. 1.**
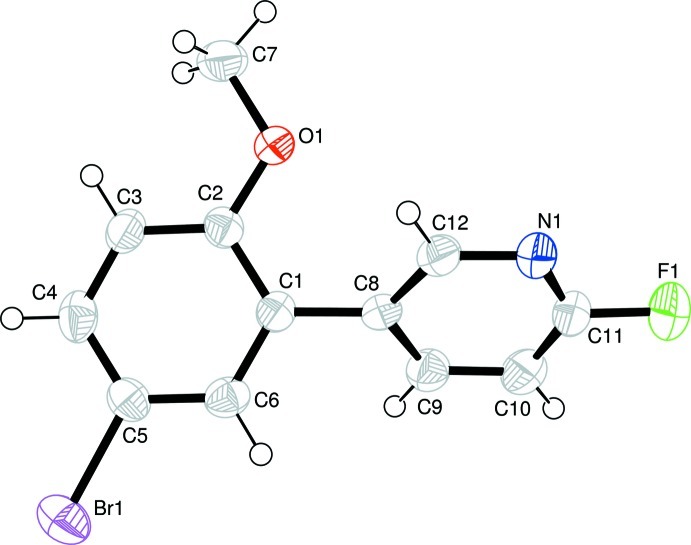
View of the title compound with displacement ellipsoids drawn at the 50% probability level.

## supplementary crystallographic information

### Comment

We have reported the crystal structure of 5-(4-chlorophenyl)-2-fluoropyridine (Adeel *et al.*, 2012), 4-(2-fluoropyridin-5-yl)phenol (Elahi *et al.*, 2012*a*), 5-(2,3-dichlorophenyl)-2-fluoropyridine (Elahi *et al.*, 2012*b*) and 2-fluoro-5-(4-fluorophenyl)pyridine (Elahi *et al.*, 2012) which have common moiety of 2-fluoropyridine as in the title compound, (Fig. 1).

In the title compound, the 1-bromo-4-methoxybenzene A (C1–C7/BR1/O1) and the 2-fluoropyridine B (C8—C12/N1/F1) systems are almost planar with r.m.s. deviations of 0.0172 Å and 0.0087 Å, respectively. The dihedral angle between A/B is 51.39 (5)°. There does not exist any kind of H-bonding and the molecules interact by van der Waals forces.

### Experimental

To a 6 ml solution of 5-bromo-2-fluoropyridine (0.20 g, 1.13 mmol), 5-bromo-2-methoxyphenylboronic acid (0.314 g, 1.36 mmol) in dioxane and K_3_PO_4_ (0.361 g, 1.7 mmol, in 1 ml H_2_O) was added Pd(PPh_3_)_4_ (1.5 mole %) at 373 K under N_2_ atmosphere. The reaction mixture was refluxed for 8 h. Then 20 ml of distilled water was added. The aqueous layer was extracted three times with EtOAc(3 × 15 ml). The organic layer was evaporated in *vacuo* and the crude product was obtained. Colorless needles of (I) were obtained by the recrystalization of crude product in a saturated CHCl_3_/CH_3_OH solution.

Yield: 0.294 g, 92%. m.p. 345–347 K.

### Refinement

The H-atoms were positioned geometrically (C–H = 0.93–0.96 Å) and refined as riding with *U*_iso_(H) = *xU*_eq_(C), where *x* = 1.5 for methyl and *x* = 1.2 for other H-atoms.

### Crystal data

**Table:**

C_12_H_9_BrFNO	*F*(000) = 560
*M**_r_* = 282.11	*D*_x_ = 1.714 Mg m^−^^3^
Monoclinic, *P*2_1_/*c*	Mo *K*α radiation, λ = 0.71073 Å
Hall symbol: -P 2ybc	Cell parameters from 1441 reflections
*a* = 3.9376 (4) Å	θ = 1.8–25.5°
*b* = 20.999 (3) Å	µ = 3.75 mm^−^^1^
*c* = 13.2700 (15) Å	*T* = 296 K
β = 95.035 (7)°	Needle, colorless
*V* = 1093.0 (2) Å^3^	0.34 × 0.18 × 0.16 mm
*Z* = 4	

### Data collection

**Table:**

Bruker Kappa APEXII CCD diffractometer	2027 independent reflections
Radiation source: fine-focus sealed tube	1441 reflections with *I* > 2σ(*I*)
Graphite monochromator	*R*_int_ = 0.042
Detector resolution: 8.10 pixels mm^-1^	θ_max_ = 25.5°, θ_min_ = 1.8°
ω scans	*h* = −4→4
Absorption correction: multi-scan (*SADABS*; Bruker, 2005)	*k* = −25→15
*T*_min_ = 0.674, *T*_max_ = 0.698	*l* = −15→16
7773 measured reflections	

### Refinement

**Table:**

Refinement on *F*^2^	Primary atom site location: structure-invariant direct methods
Least-squares matrix: full	Secondary atom site location: difference Fourier map
*R*[*F*^2^ > 2σ(*F*^2^)] = 0.035	Hydrogen site location: inferred from neighbouring sites
*wR*(*F*^2^) = 0.068	H-atom parameters constrained
*S* = 1.04	*w* = 1/[σ^2^(*F*_o_^2^) + (0.027*P*)^2^] where *P* = (*F*_o_^2^ + 2*F*_c_^2^)/3
2027 reflections	(Δ/σ)_max_ = 0.001
146 parameters	Δρ_max_ = 0.29 e Å^−^^3^
0 restraints	Δρ_min_ = −0.28 e Å^−^^3^

### Special details

**Table:**

Geometry. Bond distances, angles *etc*. have been calculated using the rounded fractional coordinates. All su's are estimated from the variances of the (full) variance-covariance matrix. The cell e.s.d.'s are taken into account in the estimation of distances, angles and torsion angles
Refinement. Refinement of *F*^2^ against ALL reflections. The weighted *R*-factor *wR* and goodness of fit *S* are based on *F*^2^, conventional *R*-factors *R* are based on *F*, with *F* set to zero for negative *F*^2^. The threshold expression of *F*^2^ > σ(*F*^2^) is used only for calculating *R*-factors(gt) *etc*. and is not relevant to the choice of reflections for refinement. *R*-factors based on *F*^2^ are statistically about twice as large as those based on *F*, and *R*- factors based on ALL data will be even larger.

### Fractional atomic coordinates and isotropic or equivalent isotropic displacement parameters (Å^2^)

**Table:**

	*x*	*y*	*z*	*U*_iso_*/*U*_eq_	
Br1	0.56028 (8)	0.42649 (2)	0.65535 (3)	0.0533 (1)	
F1	0.6238 (6)	0.43194 (10)	−0.01878 (15)	0.0815 (9)	
O1	1.0021 (5)	0.25300 (10)	0.32894 (14)	0.0422 (7)	
N1	0.5368 (7)	0.35827 (13)	0.0979 (2)	0.0501 (11)	
C1	0.7736 (7)	0.35084 (13)	0.3789 (2)	0.0326 (10)	
C2	0.9046 (7)	0.29045 (14)	0.4060 (2)	0.0337 (10)	
C3	0.9321 (7)	0.27195 (15)	0.5063 (2)	0.0407 (11)	
C4	0.8285 (7)	0.31185 (16)	0.5803 (3)	0.0439 (11)	
C5	0.7032 (7)	0.37101 (14)	0.5544 (2)	0.0377 (11)	
C6	0.6760 (7)	0.39044 (14)	0.4547 (2)	0.0378 (10)	
C7	1.1517 (8)	0.19256 (15)	0.3560 (3)	0.0511 (12)	
C8	0.7416 (7)	0.37357 (13)	0.2724 (2)	0.0333 (10)	
C9	0.8664 (7)	0.43295 (15)	0.2473 (2)	0.0429 (11)	
C10	0.8273 (8)	0.45436 (16)	0.1482 (3)	0.0510 (11)	
C11	0.6638 (9)	0.41379 (17)	0.0803 (3)	0.0516 (12)	
C12	0.5761 (7)	0.33878 (15)	0.1936 (2)	0.0418 (11)	
H3	1.02142	0.23212	0.52415	0.0488*	
H4	0.84380	0.29866	0.64746	0.0525*	
H6	0.59073	0.43076	0.43811	0.0454*	
H7A	1.23508	0.17326	0.29741	0.0763*	
H7B	0.98311	0.16542	0.38171	0.0763*	
H7C	1.33727	0.19847	0.40709	0.0763*	
H9	0.97674	0.45841	0.29733	0.0514*	
H10	0.90758	0.49388	0.12940	0.0611*	
H12	0.48683	0.29927	0.20876	0.0503*	

### Atomic displacement parameters (Å^2^)

**Table:**

	*U*^11^	*U*^22^	*U*^33^	*U*^12^	*U*^13^	*U*^23^
Br1	0.0574 (2)	0.0553 (2)	0.0484 (2)	−0.0061 (2)	0.0108 (2)	−0.0154 (2)
F1	0.138 (2)	0.0654 (14)	0.0405 (13)	0.0176 (14)	0.0042 (12)	0.0153 (12)
O1	0.0590 (13)	0.0327 (12)	0.0339 (13)	0.0100 (11)	−0.0007 (10)	0.0006 (11)
N1	0.0724 (19)	0.0421 (18)	0.0340 (18)	0.0103 (16)	−0.0060 (14)	0.0015 (15)
C1	0.0329 (16)	0.0309 (17)	0.0330 (19)	−0.0033 (14)	−0.0023 (14)	0.0005 (15)
C2	0.0343 (16)	0.0343 (17)	0.0316 (19)	−0.0043 (15)	−0.0022 (14)	−0.0027 (16)
C3	0.0501 (18)	0.0339 (18)	0.037 (2)	−0.0014 (16)	−0.0026 (15)	0.0062 (16)
C4	0.0507 (19)	0.048 (2)	0.0316 (19)	−0.0100 (17)	−0.0040 (15)	0.0046 (17)
C5	0.0379 (17)	0.0410 (19)	0.034 (2)	−0.0041 (15)	0.0014 (14)	−0.0062 (16)
C6	0.0371 (17)	0.0322 (17)	0.043 (2)	−0.0003 (15)	−0.0025 (14)	−0.0026 (17)
C7	0.059 (2)	0.040 (2)	0.053 (2)	0.0107 (17)	−0.0028 (17)	−0.0056 (18)
C8	0.0343 (16)	0.0294 (17)	0.0362 (19)	0.0064 (14)	0.0028 (14)	0.0004 (15)
C9	0.0474 (18)	0.0372 (19)	0.043 (2)	−0.0009 (16)	−0.0016 (15)	−0.0009 (18)
C10	0.065 (2)	0.0376 (19)	0.051 (2)	0.0003 (18)	0.0087 (18)	0.0078 (19)
C11	0.071 (2)	0.050 (2)	0.034 (2)	0.021 (2)	0.0063 (17)	0.0084 (19)
C12	0.0521 (19)	0.0320 (18)	0.040 (2)	0.0030 (16)	−0.0033 (16)	−0.0003 (17)

### Geometric parameters (Å, º)

**Table:**

Br1—C5	1.898 (3)	C8—C9	1.391 (4)
F1—C11	1.365 (4)	C8—C12	1.390 (4)
O1—C2	1.371 (3)	C9—C10	1.386 (5)
O1—C7	1.432 (4)	C10—C11	1.360 (5)
N1—C11	1.298 (5)	C3—H3	0.9300
N1—C12	1.330 (4)	C4—H4	0.9300
C1—C2	1.404 (4)	C6—H6	0.9300
C1—C6	1.385 (4)	C7—H7A	0.9600
C1—C8	1.487 (4)	C7—H7B	0.9600
C2—C3	1.382 (4)	C7—H7C	0.9600
C3—C4	1.380 (5)	C9—H9	0.9300
C4—C5	1.369 (4)	C10—H10	0.9300
C5—C6	1.380 (4)	C12—H12	0.9300
			
C2—O1—C7	117.2 (2)	N1—C11—C10	127.7 (4)
C11—N1—C12	115.7 (3)	N1—C12—C8	124.3 (3)
C2—C1—C6	118.4 (2)	C2—C3—H3	120.00
C2—C1—C8	122.2 (2)	C4—C3—H3	120.00
C6—C1—C8	119.5 (2)	C3—C4—H4	120.00
O1—C2—C1	116.6 (2)	C5—C4—H4	120.00
O1—C2—C3	123.6 (3)	C1—C6—H6	119.00
C1—C2—C3	119.8 (3)	C5—C6—H6	119.00
C2—C3—C4	120.8 (3)	O1—C7—H7A	109.00
C3—C4—C5	119.7 (3)	O1—C7—H7B	109.00
Br1—C5—C4	120.2 (2)	O1—C7—H7C	109.00
Br1—C5—C6	119.5 (2)	H7A—C7—H7B	109.00
C4—C5—C6	120.3 (3)	H7A—C7—H7C	109.00
C1—C6—C5	121.0 (3)	H7B—C7—H7C	109.00
C1—C8—C9	120.9 (2)	C8—C9—H9	120.00
C1—C8—C12	122.8 (2)	C10—C9—H9	120.00
C9—C8—C12	116.3 (2)	C9—C10—H10	122.00
C8—C9—C10	120.4 (3)	C11—C10—H10	122.00
C9—C10—C11	115.6 (3)	N1—C12—H12	118.00
F1—C11—N1	114.2 (3)	C8—C12—H12	118.00
F1—C11—C10	118.1 (3)		
			
C7—O1—C2—C1	177.0 (2)	O1—C2—C3—C4	−179.8 (3)
C7—O1—C2—C3	−2.4 (4)	C1—C2—C3—C4	0.8 (4)
C12—N1—C11—F1	179.1 (3)	C2—C3—C4—C5	−1.3 (4)
C12—N1—C11—C10	−0.9 (5)	C3—C4—C5—Br1	−179.9 (2)
C11—N1—C12—C8	−0.3 (5)	C3—C4—C5—C6	0.9 (4)
C6—C1—C2—O1	−179.2 (2)	Br1—C5—C6—C1	−179.1 (2)
C6—C1—C2—C3	0.2 (4)	C4—C5—C6—C1	0.1 (4)
C8—C1—C2—O1	−0.1 (4)	C1—C8—C9—C10	−178.3 (3)
C8—C1—C2—C3	179.3 (3)	C12—C8—C9—C10	−0.8 (4)
C2—C1—C6—C5	−0.6 (4)	C1—C8—C12—N1	178.6 (3)
C8—C1—C6—C5	−179.8 (3)	C9—C8—C12—N1	1.1 (4)
C2—C1—C8—C9	−130.1 (3)	C8—C9—C10—C11	−0.2 (4)
C2—C1—C8—C12	52.6 (4)	C9—C10—C11—F1	−178.8 (3)
C6—C1—C8—C9	49.0 (4)	C9—C10—C11—N1	1.2 (5)
C6—C1—C8—C12	−128.3 (3)		
